# Editorial: Cell Organelle Exploitation by Viruses During Infection

**DOI:** 10.3389/fmicb.2021.675152

**Published:** 2021-04-23

**Authors:** Parikshit Bagchi, Indranil Banerjee, Miguel A. Martín-Acebes

**Affiliations:** ^1^Department of Cell and Developmental Biology, University of Michigan Medical School, Ann Arbor, MI, United States; ^2^Cellular Virology Lab, Department of Biological Sciences, Indian Institute of Science Education and Research (IISER), Mohali, India; ^3^Department of Biotechnology, Instituto Nacional de Investigación y Tecnología Alimentaria (INIA), Madrid, Spain

**Keywords:** virus, cell, host-virus interaction, cellular organelle, virus replication cycle

As obligate intracellular pathogens, viruses co-opt numerous cellular processes to gain entry to the host cells and establish successful infection. This research topic highlights how diverse group of viruses utilize host cell organelles at different stages of their replication cycles. Establishing myriad points of contacts with the cellular factors, viruses exploit processes linked to different organelles to deliver their genome into the host cells and turning them into virus-producing units. The articles presented under this topic shed lights on how craftily viruses manipulate the cellular organelles and their associated machineries to facilitate different steps of their life cycle and spread. That viruses are dependent on host cell factors for their entry, replication, and egress, it opens up the possibility of identifying cellular proteins that can be targeted by inhibitors to block viral infections.

The first article of this topic (Liu et al.), focuses on the importance of host long non-coding RNAs (lncRNAs) in viral pathogenesis. By RNA-seq and transcriptomic analyses, the authors show how lncRNAs can be used as efficient tools for studying Porcine delta coronavirus (PDCoV) infection process and designing novel antiviral strategies. As we are currently in the middle of a deadly pandemic caused by SARS-CoV-2, the findings of this study could be applied to gain critical insights into the disease mechanism of this virus and strategizing new therapeutic interventions.

Membrane contact sites or MCS has recently emerged as an exciting field of cell biology research. How different viruses exploit these unique structures of the host cells has drawn the attention of the scientific community. The review article by discusses Lu et al. about how a cellular protein Acyl-coenzyme A binding domain containing 3 (ACBD3) interacts with the viral 3A protein (encoded by members of the *Picornaviridae* family) at the MCS, which are used by diverse viruses to ensure lipid transfer to replication organelles (ROs).

Autophagy is an important catabolic process to maintain cellular homeostasis which involves autophagosome and lysosome, two important cellular organelles. Articles by Huang et al. and Li et al. reveal how autophagy machinery is differentially regulated by two different viruses. While Huang et al. shows how red spotted grouper nervous necrosis virus (RGNNV) induces autophagy and causes cell death by lysosomal vacuolation; Li et al. demonstrates that cellular autophagy is markedly inhibited by Singapore grouper iridovirus (SGIV).

Cellular remodeling is another crucial aspect of virus-host interaction. In this research topic, while the article by shows Otulak-Kozieł et al. how a plant virus alters host cell organelles and remodels membrane structures by structural analysis of infected plant cells, findings by demonstrates Garcia et al. how Zika virus, a mosquito-borne flavivirus, reorganizes three cellular organelles—promyelocytic leukemia nuclear body (PML-NBs), mitochondria, and lipid droplets, during it course of infection.

For successful infection, viruses exploit almost every important organelle and various cellular functions of the host cell. This important aspect is elaborated by in Banerjee et al. their detailed review on herpes simplex virus or (HSV).

Mitochondria are very important cell organelles that serve as hub of power supply of a cell and involved in cellular respiration and ATP synthesis. It also maintains cellular homeostasis by regulating cellular apoptosis and autophagy. It is also reported that mitochondria play an important role in immune modulation. Given the importance of mitochondria in several critical functions of the cell, they are one of the favorite organelles exploited by many viruses to establish productive infection. Here, the review article by discusses Dutta et al. the interplay between RNA viruses and regulation of mitochondria-induced immune responses.

Finally, the review by focuses Ramdas et al. on how HIV-1 uses various cellular compartments and cellular functions during its replication cycle, starting from its entry to production of progeny virions, and their egress. The authors discuss every step of the virus replication cycle and elaborate how various functions of different cellular compartments are exploited during infection.

The contributions in this special topic highlight current advances in the field of host-virus interactions, and they more precisely elaborate on the importance of cellular organelles in different stages of infection cycle of viruses ranging from plant viruses to human viruses, including both the DNA and RNA viruses. This knowledge could be utilized in further research and to develop novel host-directed antiviral strategies. In fact, host-targeting antiviral approaches have potential benefits such as broad-spectrum activity against related viruses sharing the route of entry or appropriating the same components of cellular machineries. Targeting host factors also provide high genetic barrier against the development of resistant viruses. In summary, the findings presented here could be very useful and relevant not only for boosting our knowledge of virus-host interactions, but also in our preparation to combat the ongoing pandemic and future viral threats.

## Author Contributions

PB, IB, and MM-A edited the topic and wrote the manuscript. All authors approved the submitted version.

## Conflict of Interest

The authors declare that the research was conducted in the absence of any commercial or financial relationships that could be construed as a potential conflict of interest.

